# Seroprevalence of Hepatitis B, Hepatitis C and Human Immunodeficiency Viruses among Thalassemia Patients in West North of Iran

**Published:** 2015-07-20

**Authors:** N Valizadeh, M Noroozi, S Hejazi, Sh Nateghi, A Hashemi

**Affiliations:** 1 **1.Assistant professor of Hematology/Medical Oncology, Urmia University of Medical Sciences, Emam Khomeini Hospital, Urmia, Iran.**; 2 **Assistant professor of Hematology/Medical Oncology, Tehran University of Medical Sciences, Shariati Hospital,Tehran,Iran.**; 3 **Assistant professor of Pediatric Hematology/Medical Oncology, Department of Pediatric hematology, Motahari Hospital, Urmia University of Medical Sciences, Urmia, Iran. **; 4 **Assistant professor of Pediatric Hematology/Medical Oncology, Department of pediatric hematology, Motahari Hospital, Urmia University of Medical Sciences, Urmia , Iran.**; 5 **General physician, Urmia University of Medical Sciences, Urmia, Iran.**; 6 **Assistant professor of Gastroenterology, Urmia University of Medical Sciences, Urmia, Iran.**

**Keywords:** Antibody, HBs Antigen, HCV, HIV, Thalassemia, TransfusionResults

## Abstract

**Background:**

Thalassemia patients that are conventionally treated by a regular transfusion regimen are exposed to blood born viral infections.The aim of this study was to investigate the seroprevalence of hepatitis B virus (HBV), hepatitis C virus (HCV), and human Immunodeficiency virus (HIV) among all multitransfused thalassemia patients in west north of Iran.

**Material and Methods:**

A retrospective study was conducted in February 2014, on 32 patients in Urmia, suffering from transfusion dependent thalassemia were admitted to Motahari and Emam Khomeini hospitals. Patients’ medical records were studied for HBs antigen and seropositivity for HCV, and HIV antibodies.

**Results:**

Out of 32transfusion dependent thalassemia patients aged between 5-17years, 18 (56.25%) and14 (43.75%) were male and female, respectively. All of them were found seronegative for HBs antigen, HCV, and HIV antibodies.

**Conclusion:**

It seems that screening of blood products is efficient in Urmia, capital of West Azarbaijan, Iran for prevention of blood born viral infections.

## Introduction

Thalassemia is a form of hereditary anemia. Patients with thalassemia have deficiency in hemoglobin production and mild or severe anemia that lead to inappropriate oxygen transport to body tissues (1).

Iran is situated in the middle of thalassemia belt and has a high frequency rate for thalassemia carrier(2).More than 25,000 cases of thalassemia major are reported in Iran(3).

Although regular blood transfusion in patients with thalassemia has improved their overall survival and quality of life, but it can increase risk of transmission of blood born viral infections, especially viral hepatitis (4-6). A study on transfusion dependent thalassemia patients in Iran showed that the rate of hepatitis C infection was higher than hepatitis B infection (7). Despite recent success in screening of blood products, hepatitis C infection is remained as an important issue in transfusion dependent thalassemia patients (8-9). In a study conducted on 50 children with transfusion dependent thalassemia, 20% of participants were positive for hepatitis B , and 30%for hepatitis C infection(10). Mohamed R, et al. studied on prevalence of hepatitis C 

infection among children with β-thalassemia major in Mid Delta, Egypt , and concluded that all participants were negative for HBsAg. HCV Ab by enzyme linked immunosorbant assay (ELISA) was positive in 76%, negative in 20%, and equivocal in 4% of participants. 40% of them had positive PCR for HCV (11).

Another study was conducted by Rehman M, et al. on transfusion dependent thalassemia patients in Pakistan and showed a HCV infection in 35%, hepatitis B infection in 1.7%, and HIV seropositivity in none of patients (12).

Prevalence of HCV infection in transfusion dependent thalassemia patients in Kuwait was also 33 %( 13).

We aimed to determine the seroprevalence of HBV, HCV, and HIV infections among multitransfused thalassemia patients in Urmia, capital of West Azarbaijan, Iran.

Material and methods: This retrospective study was done in February2014 in Urmia, Iran, on 32 transfusion dependent thalassemia patients who were admitted to Motahari and Emam Khomeini hospitals. Medical records of patients were searched for HBs Ag, HCV antibody, and HIV antibodies. Patients had been tested for HBs Ag (HBs Ag Liaison S. P. A, Italy), human immuno¬deficiency virus (HIV) Ab, and hepatitis C virus Ab with enzyme linked immunosorbant assay (HCV& HIV Ab: Medical Biological Service, Milano Italy).This study is approved in ethical committee of Urmia University of Medical Sciences. All information from medical records of patients is kept confidentially.

## Results

All 32patients with thalassemia major and intermedia, aged between 5 to17 under regular blood transfusions were included. Out of all 32patients 18(56.25%) and14 (43.75%) were male and female, respectively. Mean age of patients was 11.41±3.181years old. Mean value of ferritin level of patients was 1598.69±605.174 ng/ml.Antibody to hepatitis B surface antigen (HBs Ab) levels ≥ 10 International Units/liter (IU/L) were considered protective. In 2 patients, HBs antibody titer was less than 10 IU/L (non protective) and for the rests (30 patients) was greater than 10 IU/L (protective). 

All of them were seronegative for HBs antigen, HCV antibody, and HIV antibodies.

## Discussion

In a study conducted by Ghafourian M, et al, on 206 thalassemia patients who were admitted to the Research Center of Thalassemia and Hemoglobinopathy of Ahvaz Shafa Hospital from March 2006 to April 2007, the overall prevalence rate of anti-HCV was 28.1%(14).In Arabic countries, prevalence of HCV infection in thalassemia patients ranges between 33 to 67.3% (13), (15), (16).

The prevalence of hepatitis B infection association with transfusion was 0.57% in reports from England in 1991–1997(17).In a study conducted by Hussain H, et al. in Pakistanfrom January 2002 to December 2003,180 β- thalassemia major children were enrolled and out of them, 75 (41.7%) children were hepatitis C positive (18).

Another study in Isfahan on 466 patients with major thalassemia during 1996-2011, was done and the prevalence of HCV was estimated 8% (19).

Shaker O, et al. studied for occult hepatitis B in Eighty Egyptian thalassemia children and found seropositivity for HCV , and HBV at 25% and 32.5%.(20). Bhavsar H, et al. studied the prevalence of HIV, hepatitis B, and hepatitis C infection in thalassemia major patients in tertiary care hospital, Gujarat, and found that out of 100 patients 18 (18%) were Anti HCV Ab positive, 6 (6%) were HBs Ag positive, and 9(9%) were anti HIV 1or 2 positive(21). Studies from India have reported that HIV seropositivity varies from 0 to% 9.3 in multi transfused thalassemia children (22-23).

 In another study from India, thirteen cases (2.8%) reported positive for HBsAg by ELISA, 107 (23.1%) reactive for anti HCV, and 11 (2.38%) for anti HIV antibodies among 462 multitransfused thalassemia patients (24).

Tamaddoni A, et al. studied for seroprevalence of HCV antibody among

Patients with β-Thalassemia major in Amirkola thalassemia Center located in Babol, Iran, on 64 females and 49 males. In their study; twelve (10.6%) cases were positive for anti-HCV antibody (25).

Ansari S studied for seropositivity of hepatitis C, hepatitis B, and HIV in chronically transfused β-thalassemia major patients in Pakistan and found that out of 160 patients, 21 cases (13.1%) were anti-HCV positive, and 2 (1.25%) were HBs Ag positive. However, HIV antibodies were not detected in any of participants (26). In this study all of 36 transfusion dependent thalassemia patients in Urmia were found seronegative for HBs Ag, HCV antibody, and HIV antibodies.

These results may be due to good quality of screening methods of blood products in Urmia. However, in comparison with other similar studies, the number of patients was fewer because the rate of thalassemia patients in West Azarbaijan, Iran was low.

## Conclusion

All transfusion dependent patients with thalassemia major and intermedia in Urmia, the capital of West Azarbaijan, were seronegative for hepatitis B, hepatitis C, and human immunodeficiency viruses. It seems that screening of blood products is efficient in Urmia for prevention of blood born viral infections. 
